# Factors Associated with Metabolic Syndrome and Related Medical Costs by the Scale of Enterprise in Korea

**DOI:** 10.1186/2052-4374-25-23

**Published:** 2013-10-21

**Authors:** Hyung-Sik Kong, Kang-Sook Lee, Eun-shil Yim, Seon-Young Lee, Hyun-Young Cho, Bin Na Lee, Jee Young Park

**Affiliations:** 1 Graduate School, The Catholic University of Korea, Seoul, Korea; 2 Address: Department of Preventive Medicine, College of Medicine, The Catholic University of Korea, Banpodae-ro 222, Seocho-gu, Seoul, Korea; 3 Department of Nursing, The Daegu Health College, Daegu, Korea

**Keywords:** Worker, Metabolic syndrome, Medical cost, Scale of enterprise

## Abstract

**Objectives:**

The purpose of this study was to identify the risk factors of metabolic syndrome (MS) and to analyze the relationship between the risk factors of MS and medical cost of major diseases related to MS in Korean workers, according to the scale of the enterprise.

**Methods:**

Data was obtained from annual physical examinations, health insurance qualification and premiums, and health insurance benefits of 4,094,217 male and female workers who underwent medical examinations provided by the National Health Insurance Corporation in 2009. Logistic regression analyses were used to the identify risk factors of MS and multiple regression was used to find factors associated with medical expenditures due to major diseases related to MS.

**Result:**

The study found that low-income workers were more likely to work in small-scale enterprises. The prevalence rate of MS in males and females, respectively, was 17.2% and 9.4% in small-scale enterprises, 15.9% and 8.9% in medium-scale enterprises, and 15.9% and 5.5% in large-scale enterprises. The risks of MS increased with age, lower income status, and smoking in small-scale enterprise workers. The medical costs increased in workers with old age and past smoking history. There was also a gender difference in the pattern of medical expenditures related to MS.

**Conclusions:**

Health promotion programs to manage metabolic syndrome should be developed to focus on workers who smoke, drink, and do little exercise in small scale enterprises.

## Introduction

The factors associated with cardiovascular diseases, such as strokes and heart disease, are known to include hypertension and hyperlipidemia, and clustering of such factors in an individual known as metabolic syndrome (MS) [1,2]. The risk of developing cardiovascular disease, such as strokes and heart disease, in MS patients was 2–5 times higher than in those without MS, and the death rate is also higher [3]; moreover, the rates of having diseases, such as diabetes, a fatty liver, polycystic ovary syndrome, chronic renal failure, severe obstructive sleep apnea, and gout are higher [4,5].

Having MS affects medical costs, and Fitch et al [6] observed that it costs US $295 more per month compared to not having MS. No data on the costs of abdominal obesity in particular, which is one of the MS factors, are available; however, the overall medical costs for overweight people with hypertension, diabetes, heart disease, stroke, and hyperlipidemia was US $10,000-15,000 more than for normal weight people [7]. Moreover, as people with diabetes spend three or more times as much as other individuals on health care [8], it is clear that MS has a great influence on US medical expenditures.

In South Korea, health insurance benefits grew 1.6 times from 16.2 trillion won in 2004 to 26.6 trillion won in 2008, and individual benefits grew from 344,000 won to 555,000 won (1,000 won is approximately 1 US dollar). Cardiac disorder and cerebrovascular patients having MS factors such as hypertensive and diabetes grew 1.3 times, from 671 million in 2004 to 890 million in 2008, and the cost of health insurance grew 1.7 times, from 1.5692 trillion won to 2.6899 trillion won [9]. Likewise, the necessity for research on patients with MS factors and increases in medical cost has been highly emphasized.

The factors of MS resulted not only in expenditures for drug coverage and direct medical costs, but also a decrease in productivity rate due to worker’s absenteeism, disability, and presenteeism related to health issues [10]. In South Korea; in 2008, out of a total of 9,734 workers who reported job-related diseases, 12% of them (1,207 workers) had cardiovascular diseases, and 482 (49%) of 974 deaths due to job-related diseases were cardiovascular disease deaths [11]. Management of MS, as a factor contributing to occupational cardiovascular disease, has been suggested to be a very important approach to reduction of both direct and indirect costs.

As of 2008, out of 3.26 million enterprises in South Korea, 98.8% of enterprises were small-scale enterprises with under 50 workers, 11.1% medium-scale enterprises with 50–300 workers, and 0.1% were large-scale enterprises with over 300 workers; however, 13.4% of all workers were employed by large-scale enterprises, 20% by medium-scale ones, and 60.6% by small-scale enterprises [12]. Given that small-scale enterprises were the vast majority of companies, most businesses had very little systematic management of health systems, given their lack of a direct relation with business. In addition, among workers, many were physically vulnerable in older age. From an analysis of worker’s physical examination data in 2007, 31.5% of workers in medium-scale and large-scale enterprises had diseases, but 37% of workers in small-scale enterprises did; notably, workers with hypertension, a MS factor comprised 38.4% out of 86,208 workers in small-scale enterprises, and 35.3% out of 48,486 workers were hyperlipidemia patients, which was higher than the rate in other sizes of enterprises [13]. From the physical examination records, 46.6% of workers with diseases did not go to clinics [14] therefore, the system of follow-up on workers’ physical examinations should be strengthened.

In the United States, most workers had covered their medical costs by their employers. Schultz and Edington [15,16] researched the economic losses caused by productivity losses and medical costs for employed people in the manufacturing industry and financial institutions; accordingly, the United States stated that they had detected industry workers’ risk factors early and implemented a health promotion program to decrease the factors. However, in South Korea, little is known about industry workers’ MS prevalence and the medical costs associated with diseases related to MS, and the health management system is missing a necessary program for industrial workers.

The aim of this study was to examine the factors influencing the MS prevalence by the scale of enterprise and medical costs of major MS factors such as heart disease, hypertension, stroke, and hyperlipidemia according to workplace characteristics in South Korea.

## Materials and methods

### Research subject

**Figure 1:**
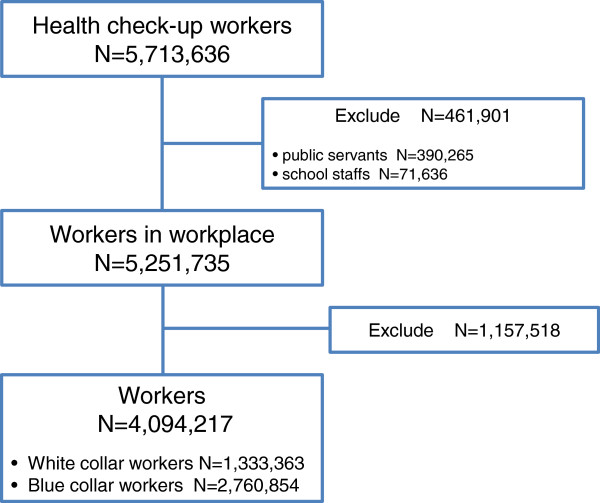
Study subjects.

The study included 5,251,735 workers at workplaces excluding 390,265 public servants and 71,636 school staffs members out of 5,713,636 health check-ups performed by the National Health Insurance Corporation from January to December in 2009. 4,094,217 workers (2,825,739 male, 1,268,478 female) classified as either white collar or blue collar workers were selected as final analysis subjects, and 1,157,518 workers who were not classified as either white collar or blue collar workers were excluded (Figure 1). The study was performed after approval by the IRB (IRB, Institutional Research Ethics Committee, confirmation number:-CUMC10U061).

### Research methods

#### Classification of workplace size

The workplaces were classified into three groups by the standards of the Occupational Safety and Health Law. Workplaces with less than 50 workers were classified as small-scale enterprises, those with more than 50 and less than 300 workers were considered medium-scale enterprises, and those with more than 300 workers were considered large-scale enterprises.

#### Lifestyle assessment

For the lifestyle assessment, a health examination questionnaire inquired about smoking, drinking, and exercise [17].

(1) Smoking: Subjects were classified as nonsmokers, past smokers, and current smokers: A nonsmoker was a person who was never exposed to smoking, a past smoker was a person who had smoked more than 5 packs (100 cigarettes) in a lifetime, and a current smoker was considered a person who smoked at the time of the study.

(2) Drinking: Drinking was classified by the number of drinks, with a nondrinker considered to be one who drank less than 1 time per a week, and others classified into those who drank 1–2 times a week, 3–4 times a week, and 5 or more times a week.

(3) Exercise Practice: Exercise practice was classified into nonactive persons, that is, who performed 30 minutes of moderate intensity exercise activity two times a week or less and active persons who exercised three times a week or more for 30 minutes each time.

#### MS diagnosis criterion

MS was evaluated by the standards of the American Heart Association/National Heart Lung, and Blood Institute (AHA/NHLBI) [18], and the waist circumference was based on the Asian-Pacific obesity standards [19]. There were five diagnosis criteria: 1) waist circumference: male ≥ 90 cm, female ≥80 cm, 2) low HDL cholesterol: HDL cholesterol male<40 mg/dL, female<50 mg/dL, 3) hypertriglyceridemia: triglycerides ≥ 150 mg/dL, 4) hypertension: systolic blood pressure/diastolic blood pressure ≥130/85 mmHg, and 5) hyperglycemia: fasting blood sugar ≥100 mg/dL, and subjects with medication for each MS risk factor were determined.

MS was defined as over three diagnosis criteria out of the five.

#### Classification of job type as white collar and blue collar

By the regulations on physical examination implemented by the Occupational Safety and Health Act enforcement regulations, a white collar worker is defined as a person who works for general affairs, human resources, sales, design, etc. in an office, not in a factory or construction area, and a person who actively works in sales and other areas is also defined as a white collar worker [20]. According to the National Health Insurance Cooperation, blue collar workers are covered for annual health examinations, and white collar workers may have an examination every two years.

#### Income level

Because employee health insurance premiums reflect a worker’s salary [9], the study used health insurance premiums as a substitute variable for the income level. In addition, the income level was classified into four quartiles of performance: first quartile (Q1), the lowest, was 1 ~ 5 quartiles (10 won ~ 29,860 won) of 20 quartiles of health insurance premium level, second quartile (Q2) was 6 ~ 10 quartiles (29,861 won ~ 49,530 won), third quartile (Q3) was 11 ~ 15 quartiles (19,531 won ~ 84,580 won), and fourth quartile (Q4), the highest, was 16 ~ 20 quartiles (84,581 won ~ 33,010,450 won).

#### Medical costs

Data was collected from the 2009 medical expenditures for cardiac disorders (G45, I20-25), stroke (I60-169), hypertension (I10-13, I15), diabetes (E10-14), and hyperlipidemia (E78), all of which are related to MS.

The 2009 medical examination data, 2009 year-end health insurance qualification and premiums data, and 2009 insurance benefits data was obtained in accordance with the regulation on private information of the National Health Insurance Cooperation with individual untraceable unique numbers for each patient. The medical examination data included MS and lifestyle factors such as waist circumference, blood sugar before a meal, HDL-cholesterol, triglycerides, blood pressure, smoking, drinking, and exercise, and qualification data included the scale of enterprise, health insurance type, and income level of insurance premiums. In addition, insurance benefits data included the medial costs of diseases except non-covered service.

#### Statistical analyses

By using SPSS 13.0 for Windows Version, a chi-squared test was conducted to analyze the differences in sociodemographic characteristics, lifestyle, and MS prevalence according to the scale of enterprise, and ANOVA analysis was conducted to analyze the medical expenditure differences of the major diseases related to MS by the scale of enterprise. A single logistic regression analysis and multiple logistic regression were used to determine the presence of any risk factors for MS, and a factor that affected medical expenditures for major diseases related to MS, that is, cardiac disorder, stroke, diabetes, and hyperlipidemia, were identified.

Model 1 analyzed the effect on medical expenditure of major diseases according to MS, and Model 2 was a multiple logistic regression according to the enterprise scale, white collar and blue collar status, general characteristics, and lifestyle factors as control variables. The reference groups selected were small-scale enterprise for enterprise scale, white collar for work type, Q1, which was the bottom 25%, for income level, nonsmoker for smoking status, and inactive for exercise performance.

## Result

### General characteristics by the scale of enterprise

For male workers, the blue collar workers were more numerous in the large-scale enterprises, at 76.5% of all large-scale enterprise workers, and the white collar workers more numerous in the small-scale enterprises, at 38.8% of all small-scale enterprise workers. For the income level, the fourth quartile, the highest income, was the most common, representing 76.5% of the workers in the large-scale enterprises, and the first quartile income, the lowest income, was the greatest, at 20.7%, in the small-scale enterprises. With regard to smoking status, 51.8% of workers in medium-scale enterprises were current smokers, 49.8% in small-scale and 43.9% in large-scale enterprises. Drinkers who drank five or more times a week comprised 9.1% of small-scale enterprise workers, 6.9% in medium-scale businesses, and 5.1% in large-scale enterprises. Those with an active lifestyle, who exercised three or more times a week, were most common, at 35.3%, in the large-scale enterprises. With regards to MS, 17.2% of the workers in the small-scale and 15.9% in the medium-scale and large-scale enterprises had three or more MS factors. Among the female workers, blue collar workers were more common in the large-scale enterprises, at 69.1%, than the other sizes of enterprises, and white collar workers were a larger proportion of workers in the in small-scale enterprises, at 49.0%. For the income level, the first quartile, the lowest income, was the highest, at 49.9%, in the small-scale enterprises, and the fourth quartile, the highest income, was the highest, at 76.5%, in the large-scale enterprises, but was only 3.5% in the small-scale enterprises. Current and past smokers were high, at 4.4% and 3.5%, in the large-scale enterprises. Moreover, drinkers who drank more than five times a week were also the highest in the large-scale industries, at 1.5%. Those with an active lifestyle were 25.4% in small-scale enterprises. Those with three or more MS factors were 9.4% of workers in small-scale, 8.9% in medium-scale, and 5.5% in large-scale enterprises (Table 1).

**Table 1:** General characteristics and health behaviors of workers by the scale of enterprise %

**Variables**	**Male**			**Female**		
	**<50**	**50-299**	**300≤**	**<50**	**50-299**	**300≤**
	**n = 1,002,756**	**n = 757,923**	**n = 1,065,060**	**n = 534,410**	**n = 309,725**	**n = 424,343**
Job						
Blue collar	61.2	73.8	76.5	51.0	66.6	69.1
Age (years)						
Mean ± SD	42.5 ± 11.4	40.7 ± 11.1	40.0 ± 9.6	39.7 ± 11.1	39.7 ± 11.6	34.7 ± 10.6
Insurance premiums						
Q4	15.5	25.9	68.3	3.5	5.3	22.3
Q3	32.8	38.6	20.0	11.3	22.0	31.9
Q2	31.0	20.0	7.4	35.2	41.9	24.7
Q1	20.7	15.5	4.4^*^	49.9	30.9	21.1^*^
Smoking						
Non-smoker	27.5	26.6	30.5^*^	95.6	95.3	92.0^*^
Ex-smoker	22.7	21.6	25.6	1.9	1.9	3.5
Current	49.8	51.8	43.9	2.5	2.8	4.4
Alcohol consumption						
Non-drinker	0.4	1.0	0.8^*^	0.7	1.6	1.9^*^
1-2/wk	58.0	59.6	59.5	89.0	87.3	84.4
3-4/wk	32.5	32.6	34.5	9.2	10.0	12.2
≥5/wk	9.1	6.9	5.1	1.1	1.1	1.5
Exercise						
≤3/wk	29.0	31.1	35.3	25.4	24.0	22.9
No. of metabolic syndrome factors						
0	29.3	31.5	32.9^*^	46.9	48.7	58.8^*^
1	30.9	30.9	30.5	29.5	28.8	25.8
2	22.6	21.7	20.6	14.2	13.7	9.9
3	12.2	11.4	11.6	6.5	6.2	4.0
4	4.4	3.9	3.7	2.4	2.3	1.3
5	0.6	0.6	0.5	0.5	0.5	0.3
Metabolic syndrome	17.2	15.9	15.9	9.4	8.9	5.5

^*^ P < 0.001.

### Factors of MS

For factors of MS in both males and females, the adjusted odds ratios were significantly higher in small-scale enterprises than large-scale ones, with age, for the lowest income workers compared with the highest income quartile, for current smokers compared with nonsmokers, and for drinkers compared with nondrinkers (Table 2).

**Table 2:** Metabolic syndrome factors among workers

**Variables**	**Categories**	**Metabolic syndrome (ref. no MS)**			
		**Male**		**Female**	
		**Crude OR**^**† **^**(95% CI**^**‡**^**)**	**Adjusted OR**^**† **^**(95% CI**^**‡**^**)**	**Crude OR**^**† **^**(95% CI**^**‡**^**)**	**Adjusted OR**^**† **^**(95% CI**^**‡**^**)**
Size	≥300	1	1	1	1
	50-299	1.0(0.99–1.01)	1.01(1.00–1.02)	1.67(1.64–1.71)	1.12(1.10–1.14)
	<50	1.1(1.09–1.10)	1.05(1.04–1.06)	1.78(1.75–1.81)	1.20(1.18–1.22)
Job	White collar workers	1	1	1	1
	Blue collar workers	1.08(1.07–1.09)	0.99(0.98–1.00)	1.52(1.49–1.54)	1.08(1.07–1.10)
Age (years)	≤29	1	1	1	1
	30–39	2.19(2.16–2.22)	2.10(2.07–2.13)	2.44(2.37–2.51)	2.44(2.37–2.52)
	40–49	3.58(3.53–3.63)	3.30(3.25–3.35)	5.61(5.47–5.76)	5.19(5.05–5.34)
	50–59	4.44(4.38–4.50)	4.17(4.11–4.23)	13.28(12.94–13.63)	12.11(11.78–12.45)
	≥60	5.09(5.00–5.17)	4.86(4.77–4.94)	27.22(26.40–28.07)	23.88(23.12–24.66)
Insurance premiums	Q4	1	1	1	1
	Q3	0.77(0.76–0.78)	0.87(0.87–0.88)	0.93(0.90–0.95)	1.04(1.01–1.08)
	Q2	0.72(0.71–0.72)	0.84(0.83–0.85)	1.37(1.34–1.41)	1.17(1.13–1.20)
	Q1	1.12(1.11–1.13)	1.02(1.01–1.03)	2.62(2.55–2.69)	1.34(1.30–1.37)
Smoking	Non-smoker	1	1	1	1
	Ex-smoker	1.41(1.40–1.43)	1.20(1.19–1.21)	0.60(0.57–0.63)	1.24(1.17–1.30)
	Current	1.23(1.22–1.24)	1.26(1.25–1.27)	0.79(0.76–0.82)	1.49(1.43–1.56)
Alcohol consumption	Non-drinker	1	1	1	1
	1–2/wk	1.20(1.15–1.25)	1.13(1.09–1.18)	1.23(1.16–1.31)	1.06(1.00–1.13)
	3–4/wk	1.55(1.49–1.62)	1.41(1.35–1.47)	0.79(0.74–0.84)	1.04(0.97–1.11)
	≥5/wk	1.87(1.79–1.96)	1.49(1.42–1.55)	1.05(0.96–1.14)	1.09(1.00–1.19)
Exercise	≥3/wk	1	1	1	1
	≤2/week	1.07(1.07–1.08)	1.01(1.01–1.02)	1.06(1.04–1.07)	1.00(0.99–1.02)

^†^OR: odds ratio.

^‡^CI: confidence interval.

### Medical costs of major diseases by scale of enterprise

Among the male workers, the medical costs for small-scale enterprise workers either with MS or not were significantly higher among both white collar and blue collar workers, those aged over 60 years, those in the bottom two income quartiles, nonsmokers, past smokers, drinkers, and those with an active lifestyle. In all of the enterprises, the medical costs of major diseases for those with MS were 2 to 6 times higher than for those without MS. Among those with MS, the expenditures for medical costs for the top income quartile workers and nondrinkers in small-scale enterprises was up to 1.7 times higher than among the same workers in large-scale enterprise.

For the female workers, the medical costs for small-scale enterprise workers with MS were significantly higher among the blue collar workers, those aged under 29, aged between 50 and 59, and aged over 60 years, those in the Q2-Q4 income quartiles, current smokers, nondrinkers, and drinkers who drank 3–4 times a week and 5 or more times a week. In all types of enterprises, the medical costs of major diseases in those with MS were 2 to 16 times higher than in those without MS. In those with MS, the expenditures for the medical costs for those in the top income quartile in small-scale enterprises was 2.7 times higher than for the equivalent group in large-scale enterprises (Table 3).

**Table 3:** **Medical costs of major diseases**^**† **^**among workers by scale of enterprise unit = 1,000 KRW**

**Variables**		**Male**					**Female**			
	**Metabolic syndrome**	**<50**	**50–299**	**≥300**	**p-value**	**Metabolic syndrome**	**<50**	**50–299**	**≥300**	**p-value**
		**Mean**	**Mean**	**Mean**			**Mean**	**Mean**	**Mean**	
Job										
White collar	No	57	42	39	0.001	No	19	17	11	0.001
	Yes	241	201	181	0.001	Yes	212	216	163	0.001
Blue collar	No	67	58	49	0.001	No	38	39	20	0.001
	Yes	251	240	172	0.001	Yes	263	255	206	0.001
Age (years)										
≤29	No	2	2	2	0.188	No	1	1	1	0.460
	Yes	14	19	15	0.153	Yes	22	14	15	0.044
30–39	No	11	11	12	0.001	No	5	4	4	0.185
	Yes	63	66	63	0.063	Yes	56	56	44	0.022
40–49	No	57	58	60	0.001	No	30	33	26	0.000
	Yes	204	205	167	0.001	Yes	176	182	157	0.001
50–59	No	140	152	143	0.001	No	90	96	99	0.001
	Yes	382	383	336	0.001	Yes	320	307	305	0.023
≥60	No	262	244	247	0.001	No	188	178	179	0.145
	Yes	558	507	498	0.001	Yes	449	405	407	0.001
Insurance premiums										
Q4	No	87	62	49	0.001	No	38	15	9	0.001
	Yes	288	221	165	0.001	Yes	332	180	122	0.001
Q3	No	51	38	25	0.001	No	21	17	6	0.001
	Yes	203	173	139	0.001	Yes	248	188	107	0.001
Q2	No	51	34	40	0.001	No	20	24	15	0.001
	Yes	216	183	193	0.001	Yes	217	203	163	0.001
Q1	No	84	115	124	0.001	No	36	56	47	0.001
	Yes	315	357	382	0.001	Yes	249	295	278	0.001
Smoking										
Non-smoker	No	73	60	47	0.001	No	29	32	18	0.001
	Yes	278	248	176	0.001	Yes	245	248	204	0.002
Ex-smoker	No	106	89	72	0.001	No	14	12	6	0.001
	Yes	344	309	230	0.001	Yes	194	208	106	0.001
Current	No	39	37	31	0.001	No	16	15	5	0.001
	Yes	178	185	139	0.001	Yes	189	171	87	0.001
Alcohol consumption										
Non-drinker	No	63	51	42	0.006	No	43	44	14	0.006
	Yes	250	188	144	0.014	Yes	268	209	150	0.014
1–2/wk	No	66	55	48	0.001	No	30	33	18	0.001
	Yes	264	244	178	0.001	Yes	248	252	206	0.001
3–4/wk	No	55	49	42	0.001	No	17	17	8	0.001
	Yes	221	207	164	0.001	Yes	185	184	119	0.001
≥5/wk	No	72	65	55	0.001	No	24	20	15	0.026
	Yes	252	246	201	0.001	Yes	191	167	125	0.013
Exercise										
≤2/wk	No	56	47	39	0.001	No	27	30	16	0.001
	Yes	230	217	160	0.001	Yes	244	245	194	0.001
≥3/wk	No	79	68	60	0.001	No	32	35	21	0.001
	Yes	287	260	197	0.001	Yes	243	249	205	0.001

^**†**^Major diseases medical cost: cardiac disease, stroke, hypertension, diabetes, hyperlipidermia.

### Factors influencing major diseases’ medical costs

For the male workers, it appeared that the group with MS expended 161,709 won more than the group without MS for health care expenses.

In model 2, analyzing the factors influencing major diseases’ medical costs, those with MS were found to have paid 127,868 won more than those without MS, and workers at medium-scale enterprises paid 6,664 won more and those at large-scale enterprises paid 3,994 won more than in enterprises having less than 50 workers. Similarly, the blue collar workers paid 8,653 won less than the white collar workers, and those in the second income quartile paid 11,502 won less, those in Q3 paid 19,987 won less, and those in Q4 paid 33,512 won less than those in Q1, that is, the lowest income quartile. In the case of smoking, the past smokers paid 17,209 won more and the current smokers paid 11,883 won less than the nonsmokers. As the number of drinking increased, 8,436 won of major medial expenditure decreased, and 7,610 won was increased with age. The major medical costs for a person who exercised 3 or more times a week was 11,426 won higher than for a person who exercised less than 2 times a week.

For the female workers, model 1 showed that the group with MS expended 208,067 won more, and model 2 showed that 162,257 won more was paid for a person with MS, 3,277 won more for those at medium-scale enterprises, and 7,102 won more for large-scale enterprises was expended compared with those at small-scale enterprises. Similarly, 4,048 won more was paid with increasing age. Blue collar workers paid less than white collar workers, and those in the Q2 group paid 1,635 won less, Q3 paid 1,856 won less, and Q4 paid 11,423 won less than those in Q1, the lowest income quartile. For smoking, the past smokers paid 9,434 won more and the current smokers paid 15,622 won more than the nonsmokers. As the number of drinks per week increased, major medial expenditures were decreased by 525 won, and a person who exercised 3 or more times a week had 1,945 won higher major medical costs than a person who exercised 2 times a week or less (Table 4).

**Table 4:** **Factors influencing major diseases’ medical costs**^**† **^**among workers unit = KRW**

**Variables**	**Categories**	**Males (Medical cost)**		**Females (Medical cost)**	
		**Model 1**	**Model 2**	**Model 1**	**Model 2**
		**β**	**β**	**β**	**β**
Metabolic syndrome	No				
	Yes	161,709	127,868	208,067	162,257
Size	≥300		3,994		7,102
	50-299		6,664		3,277
	<50				
Job	White collar workers				
	Blue collar workers		-8,653		-817
Age			7,610		4,048
Insurance premium	Q4		-33,512		-11,423
	Q3		-19,987		-856
	Q2		-11,502		-1,635
	Q1				
Smoking	Non-smoker				
	Ex-smoker		17,209		9,434
	Current		-11,883		5,622
Alcohol consumption			-8,436		-525
Exercise	≤2/wk				
	≥3/wk		11,428		1,945
	R	0.15	0.26	0.25	0.32
	Adj R2	0.02	0.07	0.06	0.10
	F	65404.073	16454.867	84768.717	11796.780
	p	0.001	0.001	0.001	0.001

^**†**^Major diseases considered for medical costs: cardiac disease, stroke, hypertension, diabetes, hyperlipidemia.

## Discussion

This study analyzed the differences in the prevalence of MS by the scale of enterprise among workers who underwent medical examinations covered by the National Health Insurance Cooperation and determined the MS factors and the medical costs of major diseases. The prevalence of MS among the workers was 16.4% in the males and 8.0% in the females, and the prevalence was 17.2% in the males and 9.4% in the females in the small-scale enterprises, that is, those with under 50 workers, 15.9% of the males and 5.5% of the females in the medium-scale enterprises, those with 50–299 workers, and 15.9% of the males and 5.5% of the females in the large-scale enterprises, with more than 300 workers. This showed that small-scale enterprises had a higher rate of MS than the others. The prevalence of MS found in the present study was 25.3% of males over 20 years old in 1998, 29.0% in 2001, and 24.1% in 2005, which was lower than the findings of the Korean National Health and Nutrition Examination Survey (KNHANES) [21]. It seemed that the rate was lower than the MS prevalence of KNHANES, which targeted the general public because of Healthy worker effect. The US NHANES of 2003–2006 showed that 1/3 of the nation had MS [22]. 22.6% of financial service workers and 27% of 6 companies’ white collar workers had MS [23], but Dalvia et al [24]. estimated that the MS prevalence was 18.7% out of all US workers using NHANES data. For Europe and Spain, the general MS prevalence was reported to be 25% [25], but the prevalence in workers was 10%, specifically 11.6% of males and 4.1% of females [26]. The MS prevalence of workers in South Korea was higher than in the US and Spain.

Among factors influencing MS, a smaller workplace, increased age, and past or current smoking status had higher MS prevalence. In many studies, it was known that a low income level increased the prevalence of MS disease significantly [27]. Low socioeconomic status caused psychosocial stress regarding physiological handicaps, and it increased the risk factors of occurrence such as abdominal obesity by activating the hypothalamo-pituitary-adrenal (HPA) axis [28]. Therefore, the high MS prevalence in the small-scale Korean enterprises was in accordance with the previous findings that low occupational grade groups had a relatively high MS prevalence.

For the medical costs of major diseases related to MS in both male and female workers, there were significant differences in all sections of type of workers, income level, smoking, drinking, and exercise. In the case of having MS or not, generally small-scale and medium-scale enterprises appeared to spend more for major diseases than did large-scale enterprises. Among people with MS, the medical costs for major diseases by the scale of enterprise was 288,000 won for males in the highest income quartile in small-scale enterprises, which was 1.7 times higher than the 165,000 won in large-scale enterprises. For females, it was 332,000 won in the top income quartile in small-scale enterprises, which was 2.7 times higher than the 122,000 won in large-scale enterprises. Regardless of the enterprise size, males with MS spent a minimum of 2.0 times and a maximum of 6.8 times more and females with MS spent a minimum of 2.3 and maximum of 16.4 times more than workers without MS.

Ricci and Chee [29] reported that workers with obesity in the US spent 11.7 million dollars more annually on increased absenteeism and presenteeism than workers without obesity, and Finkelstein et al [30]. claimed that obese workers spent 1.61 times more on medical costs than nonobese workers based on an analysis of the medical expenditures of full-time workers using a 2006 health panel. In comparing the annual medical costs for manufacturing workers according to MS status, they were 1.9 times higher ($4,016 vs $2,117) for those with MS, and the pharmaceutical costs were 2.1 times higher ($106 vs $59) [15]. In addition, according to Schultz and Edington’s analysis of annual medical costs for 3 years according to the factors of MS, $437 in decreasing more than 3 factors and $54 in decreasing 1–2 factors were decreased; on the other hand, $258 in increasing 1–2 factors and $1,348 in increasing more than 3 factors were increased^16)^. It was shown that people with MS expended 1.6 times or $2000 more annually, and people with diabetes paid 1.7 times or $3,400 more than those without diabetes [31]. From an analysis of the annual medical costs of hypertensive patients with MS in Germany, Spain, and Italy by Scholzeetal [32], each country’s cost was 24,427 Euros, 1,900 Euros, and 4,877 Euros, which were over 3 times more than people with hypertension only. People with MS spent $256 more per month, an extra $46 for cardiovascular disorders and an extra $213 for cardiovascular disorders and diabetes; therefore, the costs were 1.9 times higher than for people without MS [33].

This study showed that those with an active lifestyle spent more on medical costs for major diseases than those who did not exercise. By tracing Korean physical examination data for 6 years, it was noted that long-term exercise reduced salary by preventing 22% of having colorectal cancer and 12% of having diabetes and hypertension [34]; however, a Taiwanese analysis of ambulatory care spending in 2010 showed that exercise performers had higher medical expenditures in outpatient clinics than non-exercise performers [35]. Generally, the duration of exercise was an important factor for exercise, but this study limited its consideration of duration because the screening checklist for exercise was based on the previous week. For the future screening checklist, if it were to include exercise duration, it would examine the actual relationship between exercise and medical expenditures.

For the medical costs of smoking, for the males, it was reported that the current smokers paid 6.4% more and past smokers paid 16.1% [34] more in South Korea and the current and past smokers paid 16% and 15-32% more in the US [36]; however, for the annual medical costs of Japanese workers in 2002, the past smokers spent more than the nonsmokers, but the medical costs of the current smokers were lower [37]. In addition, a study in Taiwan showed that only past smokers spent more on medical costs [35]. For drinking, the medical costs of major diseases decreased as the number of drinks per week increased. In general, excessive drinking is known to increase medical costs, so it was unclear why this relationship between drinking and medical costs appeared in the US and Japan; moreover, they indicated that drinker expended lower medical cost [36,37]. There was a possibility that people with factors of MS did more exercise and less drinking to treat and prevent the progression of their disease, and it can be assumed that the duration of examination of the change in medical costs was too short for the workers in their 30s and 40s who comprised a large proportion of the study subjects. In addition, 64.2% of the study subjects with MS never underwent treatment in 2009, so this showed that more workers did not receive treatment than 54.4% [16] from the previous non-Korean study. Therefore, workers and employers should be made more aware of MS management.

Among the limitations of this study, it was difficult to accurately determine the duration and quantity of exercise, drinking, and smoking from the lifestyle factor screening checklist for the physical examination, and the medical expenditures could have been calculated as lower by using health insurance medical cost data, which included health insurance medical costs, but not self-payment. In addition, there was a limitation in the proper analyses of indirect costs incurred while workers were off work or were replaced due to any type of disease. However, in spite of these limitations, this study was significant in applying the data to understand workers’ MS prevalence by conducting blood tests that could diagnose MS during primary care to prevent cardiovascular disorders or detect them early by undergoing additional blood tests for blood sugar and lipids when workers were found to have been having health problems in their primary health examination.

Thus, the study primarily analyzed the relationship between the scale of enterprise in South Korea and workers’ MS prevalence and medical costs of major diseases related to MS, including heart disease, stroke, hypertension, diabetes, and hyperlipidemia. It was found that risk factors were more prevalent in smaller-scale enterprises, and thus more intensive management of MS in small-scale enterprise workers is needed.

## Conclusion

It was suggested that the health promotion program to manage the metabolic syndrome should be developed to focus on workers with smoking, drinking and little exercise in small scale industries.

## Competing interests

The authors declare that they have no competing interests.

## Authors’ contributions

HSK and KSL designed the study and directed its implementation, including quality assurance and control. ESY and SYL helped conduct literature review and prepare the materials and methods. HYC and BNL helped the data collection and analysis. JYP translated in English. All authors read and approved the final manuscript.

## References

[B1] Rana JS Nieuwdorp M Jukema JW Kastelein JJ Cardiovascular metabolic syndrome an interplay of obesity, inflammation, diabetes and coronary heart disease Diabetes Obes Metab 2007 25 218 232 10.1111/j.1463-1326.2006.00594.x 17391148

[B2] Batsis JA Nieto-Martinez RE Lopez-Jimenez F Metabolic syndrome: from global epidemiology to individualized medicine Clin Pharmacol Ther 2007 25 509 524 10.1038/sj.clpt.6100355 17851562

[B3] Jung HS Yun SN Occupaational health care management model in small scale enterprises J Korean Community Nurs 2001 25 3 647 660 Korean

[B4] Qiao Q Gao W Zhang L Nyamdorj R Tuomilehto J Metabolic syndrome and cardiovascular disease Ann Clin Biochem 2007 25 232 263 10.1258/000456307780480963 17456293

[B5] Pinkham CA Cumming ME Minuk H The metabolic syndrome and all-cause mortality in an insured lives population North Am Actuar J 2006 25 7 15 10.1080/10920277.2006.10597399

[B6] Fitch K Pyenson B Iwasaki K Metabolic syndrome and employer sponsored medical benefits: an actuarial analysis Value Health 2007 25 S21 S28

[B7] Thompson D Edelsberg J Colditz GA Bird AP Oster G Lifetime health and economic consequences of obesity Arch Intern Med 1999 25 2177 2183 10.1001/archinte.159.18.2177 10527295

[B8] State of Diabetes Complications in America A comprehensive report issued by the American association of clinical endocrinologists 2007 USA: AACE, GlaxoSmithKline

[B9] The National Health Insurance Corporation National health screening statistical yearbook.2009 2010 Seoul, Korea : NHIC

[B10] Malik S Wong ND Franklin SS Impact of the metabolic syndrome on mortality from coronary heart disease, cardiovascular disease, and all causes in United States adults Circulation 2004 25 1245 1250 10.1161/01.CIR.0000140677.20606.0E 15326067

[B11] The Korea Occupational Safety and Health Agency The status of industrial accidents 2008 Incheon, Korea: KOSHA

[B12] Ministry of Employment and Labor Report to the ministry of labor 2009 Korea: International Cooperation Bureau

[B13] The National Health Insurance Corporation 2007 Results of health checkup 2008 Seoul, Korea: NHIC

[B14] Han CH Kam S Park JY Health care utilization and its determinants of worker with Non-occupational diseases Korean J Occup Environ Med 1995 25 2 282 294 Korean

[B15] Schultz AB Edington DW Metabolic syndrome in a workplace: prevalence, co-morbidities, and economic impact Metab Syndr Relat Disord 2009 25 5 459 468 10.1089/met.2009.0008 19450154

[B16] Schultz AB Edington DW The association between changes in metabolic syndrome and changes in cost in a workplace population JOEM 2009 25 7 771 779 19528830 10.1097/JOM.0b013e3181a88da5

[B17] The National Health Insurance Corporation Manual of the regular health check up 2009 Seoul, Korea: NHIC

[B18] Grund SM Metabolic syndrome scientific statement by the American heart association and the national, heart, lung, and blood institute Arterioscler Thromb Vasc Biol 2005 25 2243 2244 10.1161/01.ATV.0000189155.75833.c7 16258150

[B19] Western Pacific Regional Office of the World Health Organization The international obesity task force. The Asia-pacific perspective; redefining obesity and its treatment 2000: WPRO

[B20] Ministry of Government Legislation Occupational safety and health Act 2009: Korean

[B21] Ministry of Health & Welfare Korean national health and nutrition examination surveys 2005 Osong Korea: Ministry of Health & Welfare

[B22] Ervin B Division of health and nutrition examination surveys. Centers for disease control and prevention. Prevalence of metabolic syndrome among adults 20 years of age and over, by sez, age, race and ethnicity, and body mass index: US 2003–2006 Nat Health Stat Report 2009 25 1 7 19634296

[B23] Godefroi R Klementowicz P Pepler C Lewis B McDonough K Goldberg RJ Metabolic syndrome in a screened worksite sample: prevalence and predictors Cardiol 2005 25 3 131 136 10.1159/000083439 15665535

[B24] Davila EP Florez H Fleming LE Lee DJ Goodman E LeBlanc WG Prevalence of the metabolic syndrome among US workers Diabetes Care 2010 25 22 33 10.2337/dc10-0681 PMC2963500 20585004

[B25] Alegría E Cordero A Laclaustra M Grima A León M Casasnovas JA Prevalence of metabolic syndrome in the Spanish working population: MESYAS registry Rev Esp Cardiol 2005 25 797 806 10.1157/13077231 16022811

[B26] Sánchez-Chaparro MA Calvo-Bonacho E González-Quintela A Fernández-Labandera C Cabrera M Sáinz JC Fernández-Meseguer A Occupation-related differences in the prevalence of metabolic syndrome Diabetes Care 2008 25 9 1884 1885 10.2337/dc08-0431 18753667 PMC2518364

[B27] Park HS Oh SW Cho SI Choi WH Kim YS The metabolic syndrome and associated lifestyle factors among South Korean adults Int J Epidemiol 2004 25 2 328 336 10.1093/ije/dyh032 15082635

[B28] Bjorntrop P Behavior and metabolic disease Int J Behav Med 1996 25 285 302 10.1207/s15327558ijbm0304_1 16250745

[B29] Ricci JA Chee E Lost productive time associated with excess weight in the U.S. workforce JOEM 2005 25 1227 1234 16340703 10.1097/01.jom.0000184871.20901.c3

[B30] Finkelstein EA DiBonaventura M Burgess SM Hale BC The costs of obesity in the workplace J Occup Environ Med 2010 25 10 971 976 10.1097/JOM.0b013e3181f274d2 20881629

[B31] Boudreau DM Malone DC Raebel MA Fishman PA Nichols GA Feldstein AC Health care utilization and costs by metabolic syndrome risk factors Metab Syndr Relat Disord 2009 25 4 305 314 10.1089/met.2008.0070 19558267

[B32] Scholze J Alegria E Ferri C Langham S Stevens W Jeffries D Uhl-Hochgraeber K Epidemiological and economic burden of metabolic syndrome and its consequences in patients with hypertension in Germany, Spain and Italy; a prevalence-based model BMC Publ Health 2010 25 529 541 10.1186/1471-2458-10-529 PMC2940918 20813031

[B33] Turek P Lietava J Foltan V Kosmalova V Dukat A Costs related to medical treatment for common cardiovascular risk factors Bratisl Lek Lisry 2010 25 10 535 540 21125797

[B34] Jee SH O’Donnell MP Suh I Kim IS Korea medical insurance corporation. The relationship between modifiable health risks and future medical care expenditures: the Korea Medical Insurance Corporation (KMIC) study Am J Health Promot 2001 25 4 244 255 10.4278/0890-1171-15.4.244 11349346

[B35] Lin TF Modifiable health risk factors and medical expenditures Soc Sci Med 2008 25 11 1727 1736 10.1016/j.socscimed.2008.09.010 18950919

[B36] Bland PC An L Foldes SS Garrett N Alesci NL Modifiable health behaviors and short-term medical costs among health plan members Am J Health Promot 2009 25 4 265 273 10.4278/ajhp.08042842 19288848

[B37] Lynch WD Chikamoto Y Imai K Lin TF Kenkel DS Ozminkowski RJ The association between health risks and medical expenditures in a Japanese corporation Am J Health Promot 2005 25 3 Suppl 238 248 15675538 10.4278/0890-1171-19.3s.238

